# Supplementary material for: Spatio-temporally separated cortical flows and spindle geometry establish physical asymmetry in fly neural stem cells
Source: Nat Commun. 2017 Nov 9;8:1383. doi: 10.1038/s41467-017-01391-w (PMC5680339; doi:10.1038/s41467-017-01391-w)
Supplement: Supplementary file 1 — Supplementary Information [file 41467_2017_1391_MOESM1_ESM.pdf]

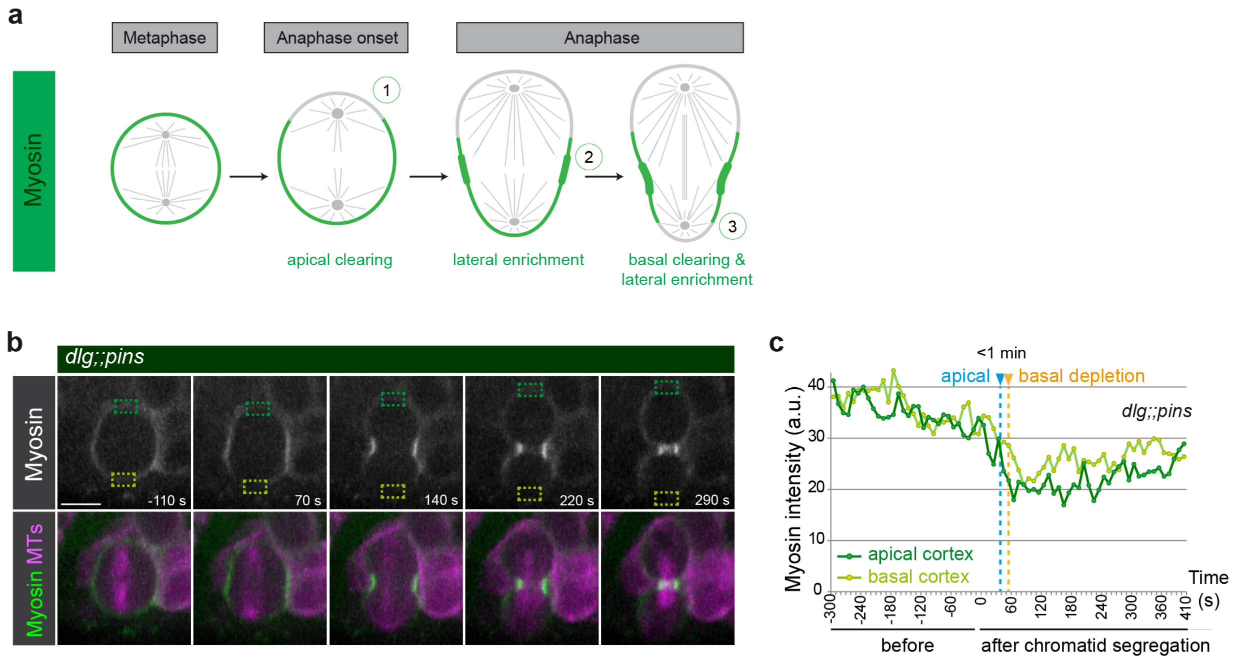


**Supplementary Figure 1: Myosin relocalization dynamics depend on neuroblast polarity** **(a)** Schematic, illustrating Myosin relocalization dynamics during the neuroblast cell cycle. **(b)** Representative image sequence of a *dlg;;pins* double mutant neuroblast, expressing Sqh:::GFP (Myosin; white on the upper panel, green in the merge) and Cherry::Jupiter (MTs; magenta in the merge). Cortical Myosin intensity was measured on the apical (dark green dashed box) and basal cortex (light green dashed box) throughout mitosis and plotted in **(c)**.

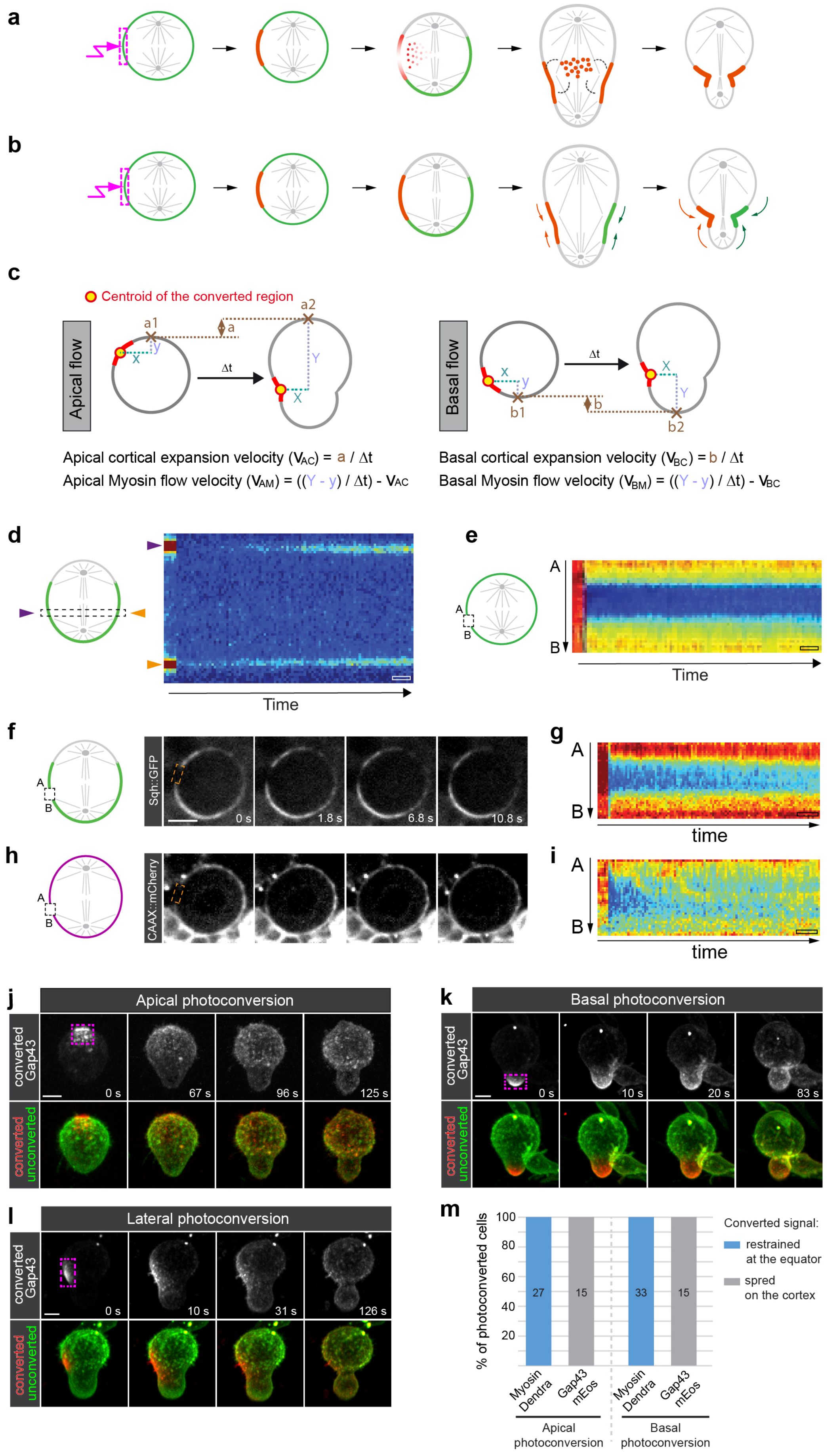

a
b
c
Centroid of the converted region
Apical flow
a1
a2
a
x
y
X
Y
Δt
Basal flow
b1
b2
b
Apical cortical expansion velocity ($V_{AC}$) = a / Δt
Apical Myosin flow velocity ($V_{AM}$) = ((Y - y) / Δt) - $V_{AC}$
Basal cortical expansion velocity ($V_{BC}$) = b / Δt
Basal Myosin flow velocity ($V_{BM}$) = ((Y - y) / Δt) - $V_{BC}$
d
Time
e
A
B
Time
f
A
B
Sqh::GFP
0 s
1.8 s
6.8 s
10.8 s
g
A
B
time
h
A
B
CAAX::mCherry
i
A
B
time
j
Apical photoconversion
converted Gap43
0 s
67 s
96 s
125 s
converted unconverted
k
Basal photoconversion
converted Gap43
0 s
10 s
20 s
83 s
converted unconverted
l
Lateral photoconversion
converted Gap43
0 s
10 s
31 s
126 s
converted unconverted
m
% of photoconverted cells
100
80
60
40
20
27
15
33
15
Myosin Dendra
Gap43 mEos
Myosin Dendra
Gap43 mEos
Apical photoconversion
Basal photoconversion
Converted signal:
restrained at the equator
spred on the cortex

**Supplementary Figure 2: Myosin flows to the cleavage furrow**

**(a, b)** Cartoons representing two possible distributions after lateral photoconversion of Myo::mDendra2. If Myosin reaches the cleavage furrow through cytoplasmic distribution, converting Myosin on the lateral cortex should result in labelling both sides of the cleavage furrow with almost equal probability. Alternatively, if Myosin flows to the cleavage furrow, the photoconverted side should remain labeled predominantly. **(c)** Cartoon representing how Myosin flow velocity was calculated. **(d)** Kymograph of a wild type anaphase neuroblast expressing Sqh::GFP performed perpendicularly to the spindle axis as represented in the cartoon. **(e)** Kymograph of a metaphase wild type, Sqh::GFP expressing, neuroblast, performed along the cortex after bleaching lateral Myosin as represented in the cartoon. Representative image sequence of a wild type neuroblast expressing **(f)** Sqh::GFP and **(h)** CAAX::mCherry (membrane associated mCherry). The bleached region is indicated with an orange dashed box. Kymographs along the bleached cortical region are shown for Sqh::GFP **(g)** and CAAX::mCherry **(i)**, respectively. Highest fluorescence intensity is shown in red, lowest in blue. **(j)** Apical, **(k)** basal and **(l)** lateral control photoconversion experiments performed in wild type neuroblasts expressing the membrane receptor Gap43 fused to the photoconvertable protein mEos (Gap43::mEos). Time is shown as seconds after the photoconversion and the purple dashed box represents the photoconverted ROI. **(m)** Quantification of the observed phenotype for the indicated markers. The number of counted cells is indicated in the bar graph. For each experiment, the data was collected from at least 3 independent experiments. For each independent experiment, at least 5 larvae were dissected.

Scale bars: 5 µm. Time scale bar (open boxes) in (d), (e), (g) and (i): 1s

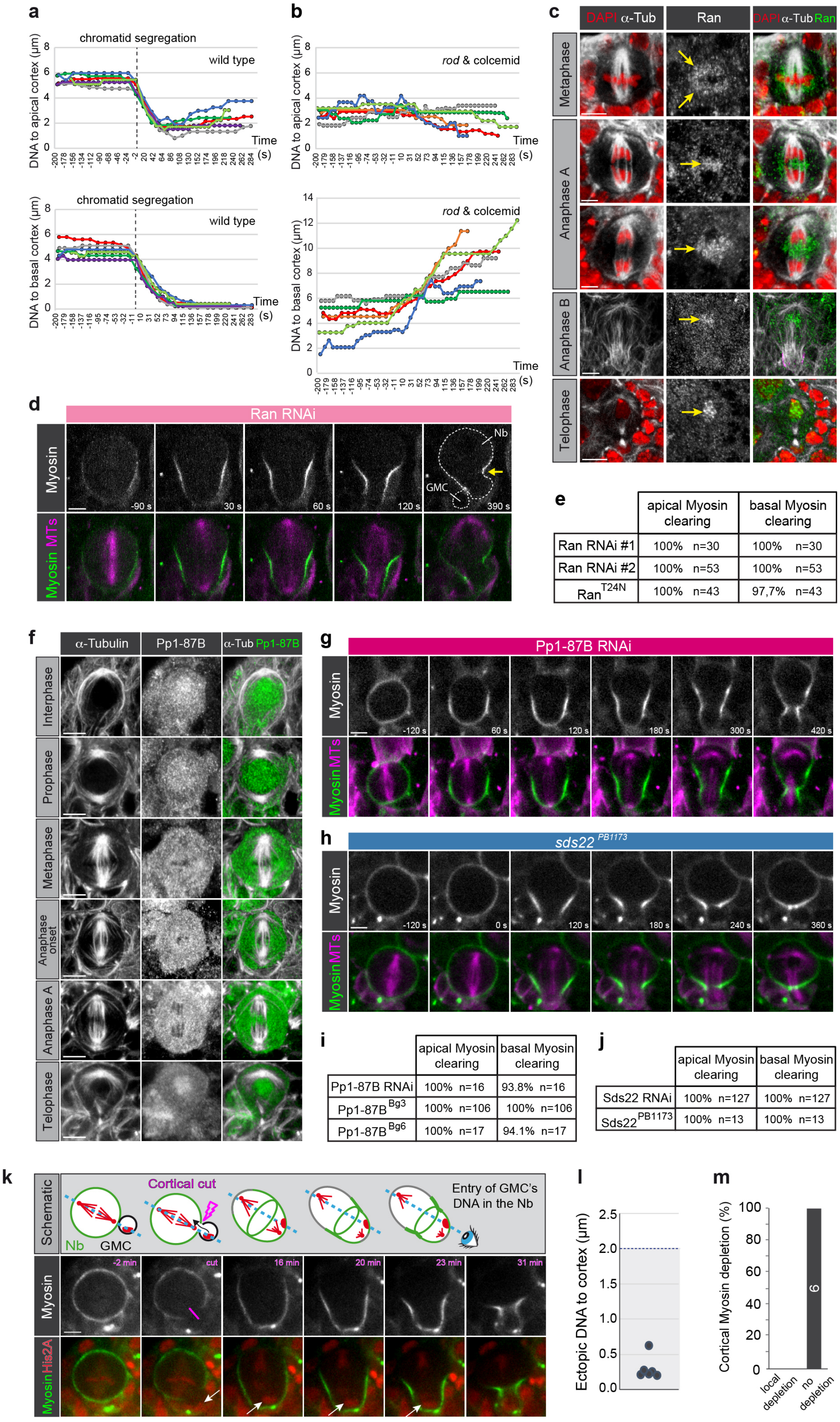


e

| | apical Myosin clearing | basal Myosin clearing |
|---|---|---|
| Ran RNAi #1 | 100% n=30 | 100% n=30 |
| Ran RNAi #2 | 100% n=53 | 100% n=53 |
| Ran$^{T24N}$ | 100% n=43 | 97,7% n=43 |

i

| | apical Myosin clearing | basal Myosin clearing |
|---|---|---|
| Pp1-87B RNAi | 100% n=16 | 93.8% n=16 |
| Pp1-87B$^{Bg3}$ | 100% n=106 | 100% n=106 |
| Pp1-87B$^{Bg6}$ | 100% n=17 | 94.1% n=17 |

j

| | apical Myosin clearing | basal Myosin clearing |
|---|---|---|
| Sds22 RNAi | 100% n=127 | 100% n=127 |
| Sds22$^{PB1173}$ | 100% n=13 | 100% n=13 |

**Supplementary Figure 3: Ran, Pp1-87B, Sds22 and GMC chromatin are not required for basal Myosin clearing.**

Plots showing the distance between chromatids and the apical (top graph) or the basal cortex (bottom graph) throughout mitosis in wild type **(a)** and colcemid-treated *rod* mutant **(b)** neuroblasts. **(c)** Dividing neuroblasts immunostained for microtubules (alpha-tubulin; white in first and third column), DNA (DAPI; red in first and third column) and Ran (white in second row; green in third column). Yellow arrows highlight Ran localization from metaphase to telophase. **(d)** Image sequence of a representative neuroblast expressing Sqh::GFP (Myosin, white; top panel, green; bottom), Cherry::Jupiter (MTs, magenta in bottom row) and RNAi against Ran. **(e)** Summary of phenotypes. Ran was knocked-down using two independent RNAi lines. $Ran^{T24N}$ is a dominant-negative. **(f)** Dividing neuroblasts immunostained for alpha-tubulin (white in first and third column) and Pp1-87B (Pp1-87B-HA; white in middle row, green in third column), from interphase to telophase. Representative Image sequences of neuroblasts expressing Sqh::GFP (white; top row, green; bottom row) and Cherry::Jupiter (magenta in overlay) and **(g)** RNAi against Pp1-87B or **(h)** mutant for *sds22* ($sds22^{PB1173}$). **(i)** Quantification of apical and basal Myosin clearing in neuroblasts lacking Pp1-87B ($Pp1\text{-}87B^{Bg3}$ and $Pp1\text{-}87B^{Bg6}$ are amorphs) or **(j)** Sds22. **(k)** Laser induced cortical cut (purple line) on a neuroblast expressing Sqh::GFP (Myosin, top row; white, bottom row; green) and His2A::mRFP (DNA, bottom row; red). **(l)** Minimal distance (µm) between the ectopic pool of DNA and the cortex, after membrane repair. Each measured cell is represented with a dot in the scatter plot. The data was collected from at least 3 independent experiments. For each independent experiment, at least 5 larvae were dissected. **(m)** Bar graph showing the percentage of wild type neuroblasts displaying cortical Myosin depletion when the proximity of the ectopic DNA with the cortex is less than 2 µm. The number of counted cells is indicated in the bar graph. Scale bars: 5 µm.

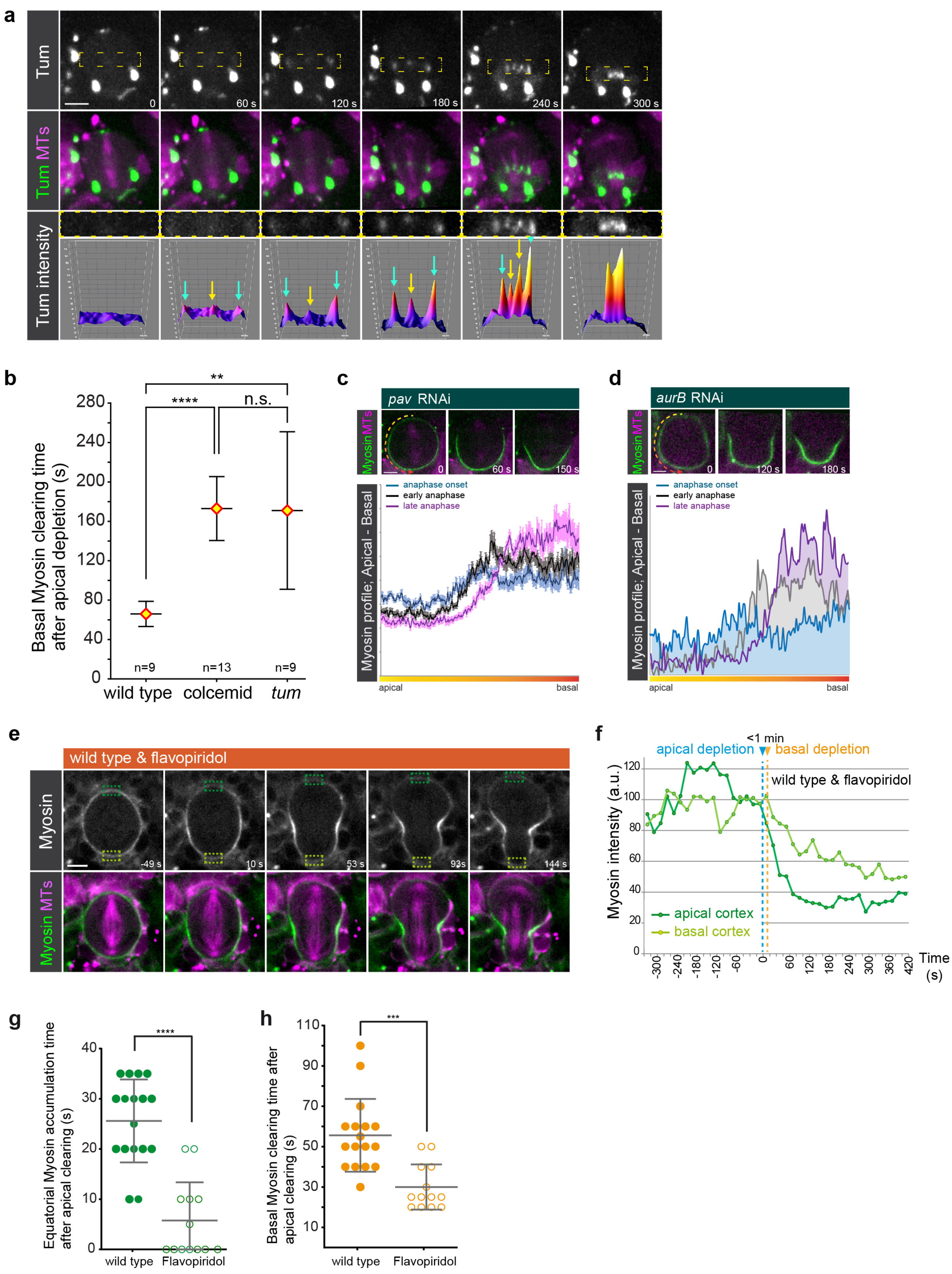

a
Tum
Tum MTs
Tum intensity
0
60 s
120 s
180 s
240 s
300 s
b
Basal Myosin clearing time after apical depletion (s)
**
****
n.s.
n=9
n=13
n=9
wild type
colcemid
tum
c
pav RNAi
Myosin MTs
0
60 s
150 s
anaphase onset
early anaphase
late anaphase
Myosin profile; Apical - Basal
apical
basal
d
aurB RNAi
Myosin MTs
0
120 s
180 s
anaphase onset
early anaphase
late anaphase
Myosin profile; Apical - Basal
apical
basal
e
wild type & flavopiridol
Myosin
Myosin MTs
-49 s
10 s
53 s
93s
144 s
f
<1 min
apical depletion
basal depletion
wild type & flavopiridol
Myosin intensity (a.u.)
apical cortex
basal cortex
Time (s)
g
Equatorial Myosin accumulation time after apical clearing (s)
****
wild type
Flavopiridol
h
Basal Myosin clearing time after apical clearing (s)
***
wild type
Flavopiridol

**Supplementary Figure 4: The centralspindlin complex is localized to the lateral neuroblast cortex, and is necessary for basal Myosin clearing.**

**(a)** Representative third instar wild type neuroblast expressing the centralspindlin component Tumbleweed fused to Venus (Tum::Venus, white; top row, green; second row) and the spindle marker Cherry::Jupiter (magenta) from anaphase onset until telophase. Third row shows a higher magnification image of the region, highlighted with the yellow dashed box. From these regions, Tum intensity was measured and plotted as a 3D graph (bottom row). Blue and yellow arrows highlight Tum accumulations at the lateral cortex and on central spindle microtubules. **(b)** Graph representing basal Myosin clearing time after apical Myosin depletion in wild-type, colcemid-treated *rod* mutant neuroblasts or after expression of RNAi against Tumbleweed. **(c)** Time-lapse of a representative third instar neuroblast, expressing Sqh::GFP (Myosin; green), Cherry::Jupiter (MTs; magenta) and RNAi against Pavarotti. Myosin intensity measurements were performed from the apical to the basal neuroblast cortex at three different time points and shown below the image sequence. Each curve represents a mean from 5 individual cells with standard deviation represented in light colors. **(d)** Time-lapse of a representative third instar neuroblast, expressing Sqh::GFP (Myosin; green), Cherry::Jupiter (MTs; magenta) and RNAi against Aurora B. Myosin intensity measurements were performed from the apical to the basal neuroblast cortex at three different time points and shown below the image sequence. **(e)** Flavopiridol-treated wild type neuroblast, expressing Sqh:::GFP (Myosin; white on the upper panel, green on the merge) and Cherry::Jupiter (MTs; magenta on the merge). Cortical Myosin intensity was measured on the apical (dark green dashed box) and basal cortex (light green dashed box) throughout mitosis and plotted in **(f)**. Equatorial Myosin accumulation **(g)** and basal **(h)** Myosin clearing time relative to apical clearing in wild type and flavopiridol-treated neuroblasts. Center values and error bars represent the mean and standard deviation (s.d), respectively. Asterisks denote statistical significance, derived from unpaired t-tests: *; $p \leq 0.05$, **; $p \leq 0.01$, ***; $p \leq 0.001$, ****; $P \leq 0.0001$, n.s.; not significant. Each measured cell (n) is represented with a dot in the scatter plots. For other graphs, the number of measured cells is indicated in the corresponding panels. For each experiment, the data was collected from at least 3 independent experiments. For each independent experiment, at least 5 larvae were dissected.

Time in seconds (s). Scale bars: 5 μm.

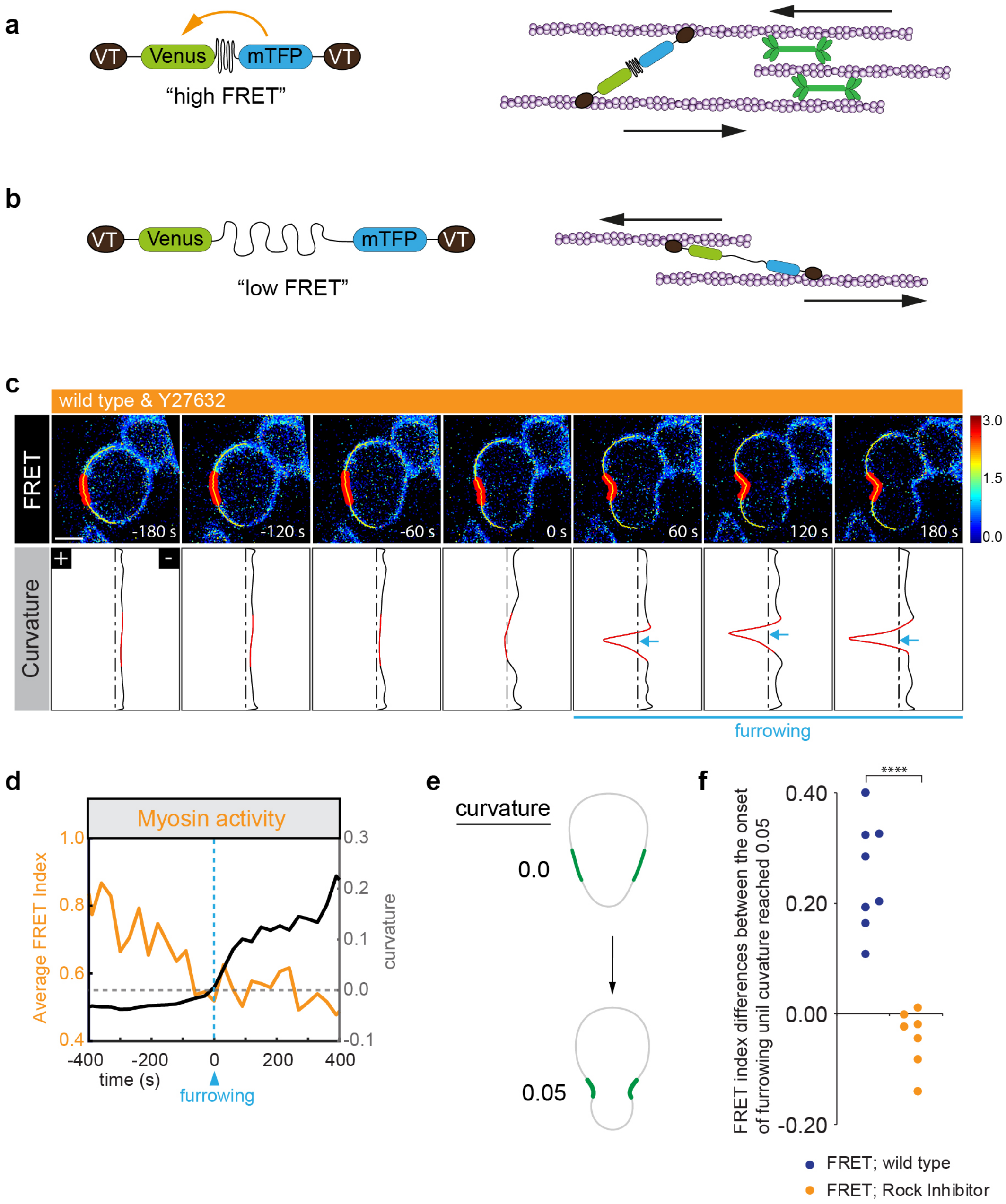


**Supplementary Figure 5: Myosin activity sensor to detect activated Myosin**

**(a)** The Myosin activity sensor is composed of a flexible spider silk protein, flanked by a FRET module, which is connected with two Vinculin (VT) domains, binding to F-Actin (see main text and methods for details and references). High FRET ratios ensue if the two vinculin domains are pushed together. **(b)** Low FRET ratios are recorded if the VT domains are pulled apart. **(c)** Representative wild type neuroblast, treated with the Rho kinase inhibitor Y27632. FRET ratios were measured along the dashed yellow line. The cleavage furrow region is highlighted in red. Curvature plots are shown underneath. **(d)** Rho kinase treated neuroblasts show a drop of activated Myosin in the furrow region (yellow line). **(e)** For wild type (blue) and Rho kinase inhibitor treated neuroblasts (yellow) the difference in FRET ratios was determined from the moment furrow ingression started (time point "0") until the curvature reached a value of "0.05". These values are plotted in **(f)**. Asterisks denote statistical significance, derived from unpaired t-tests: *;

p ≤ 0.05, **; p ≤ 0.01, ***; p ≤ 0.001, ****; P ≤ 0.0001, n.s.; not significant. Each measured cell (n) is represented with a dot in the scatter plots. The data was collected from at least 3 independent experiments. For each independent experiment, at least 5 larvae were dissected. Time in seconds (s). Scale bars: 5 μm.

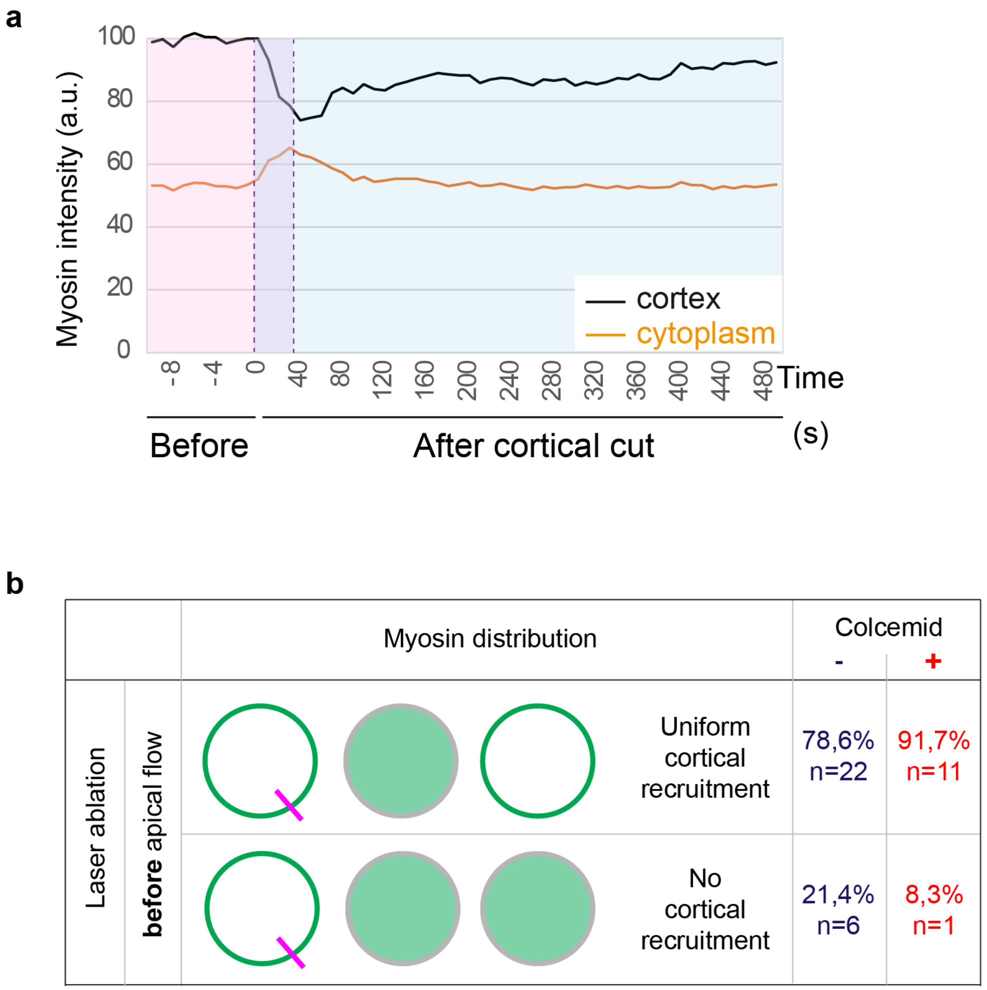


**Supplementary Figure 6: The mitotic spindle is not required for cortical Myosin recruitment in metaphase.**
**(a)** Graph showing cortical Myosin versus cytoplasmic Myosin intensity, before and after laser-induced cortical cut in metaphase. Quantifications are performed on the cell shown in Figure 6a.
**(b)** Summary of cortical cut experiments performed in metaphase.

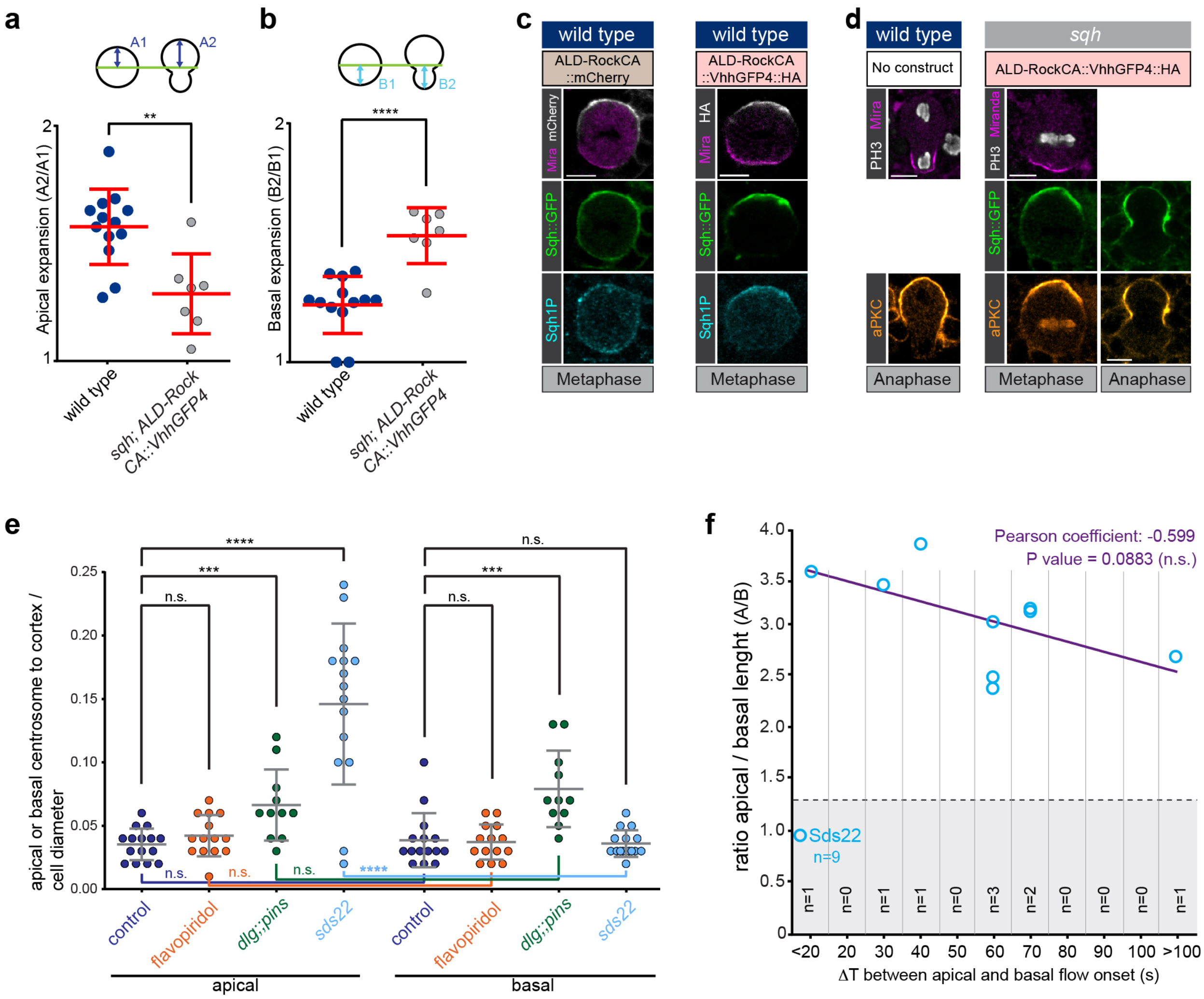


**Supplementary Figure 7: Myosin localization dynamics, spindle positioning and geometry influence sibling cell size asymmetry.**

Apical **(a)** and basal **(b)** cortical expansion for wild type neuroblasts (containing endogenous Sqh) and *sqh* mutant neuroblasts expressing the kinase domain of Rock ($Rock^{CA}$) fused to Inscuteable's apical localization domain (ALD) and the nanobody (VhhGFP4). **(c)** Representative control construct and nanobody expressing wild type neuroblast (ALD-$Rock^{CA}$-mCherry, left panel; ALD-$Rock^{CA}$::VhhGFP4-HA, right panel; endogenous Sqh still present) were stained for Miranda (purple), mCherry or HA (white), Sqh::GFP (green) and Sqh1P (blue). Localizing Rock's kinase domain to the apical cortex alone is not sufficient to enrich for phosphorylated Myosin and Sqh::GFP on the apical cortex; VhhGFP4 provides specificity by binding the GFP tag of Sqh::GFP, bringing the kinase domain in proximity of Sqh. **(d)** Representative wild type (control; left panel; endogenous Sqh still expressed) and *sqh* mutant neuroblast expressing ALD-$Rock^{CA}$::VhhGFP4-HA, stained for PH3 (white), Miranda (purple), Sqh::GFP (green) and/or aPKC (orange). **(e)** Scatter plot representing the proximity of apical and basal centrosomes to the apical and basal cortex, respectively in control, flavopiridol-treated, *dlg;;pins* and *sds22* mutant neuroblasts. **(f)** Correlation plot showing the absence of correlation between Myosin clearing on the apical and basal cortex and the asymmetry of the division, after depletion of Sds22. Center values and error bars represent the mean and standard deviation (s.d), respectively. Asterisks denote statistical significance, derived from unpaired t-tests: *; p ≤ 0.05, **; p ≤ 0.01, ***; p ≤ 0.001,

****; $P \leq 0.0001$, n.s.; not significant. Each measured cell (n) is represented with a dot in the scatter plots. For each experiment, the data was collected from at least 3 independent experiments. For each independent experiment, at least 5 larvae were dissected. Scale bars: 5 μm.

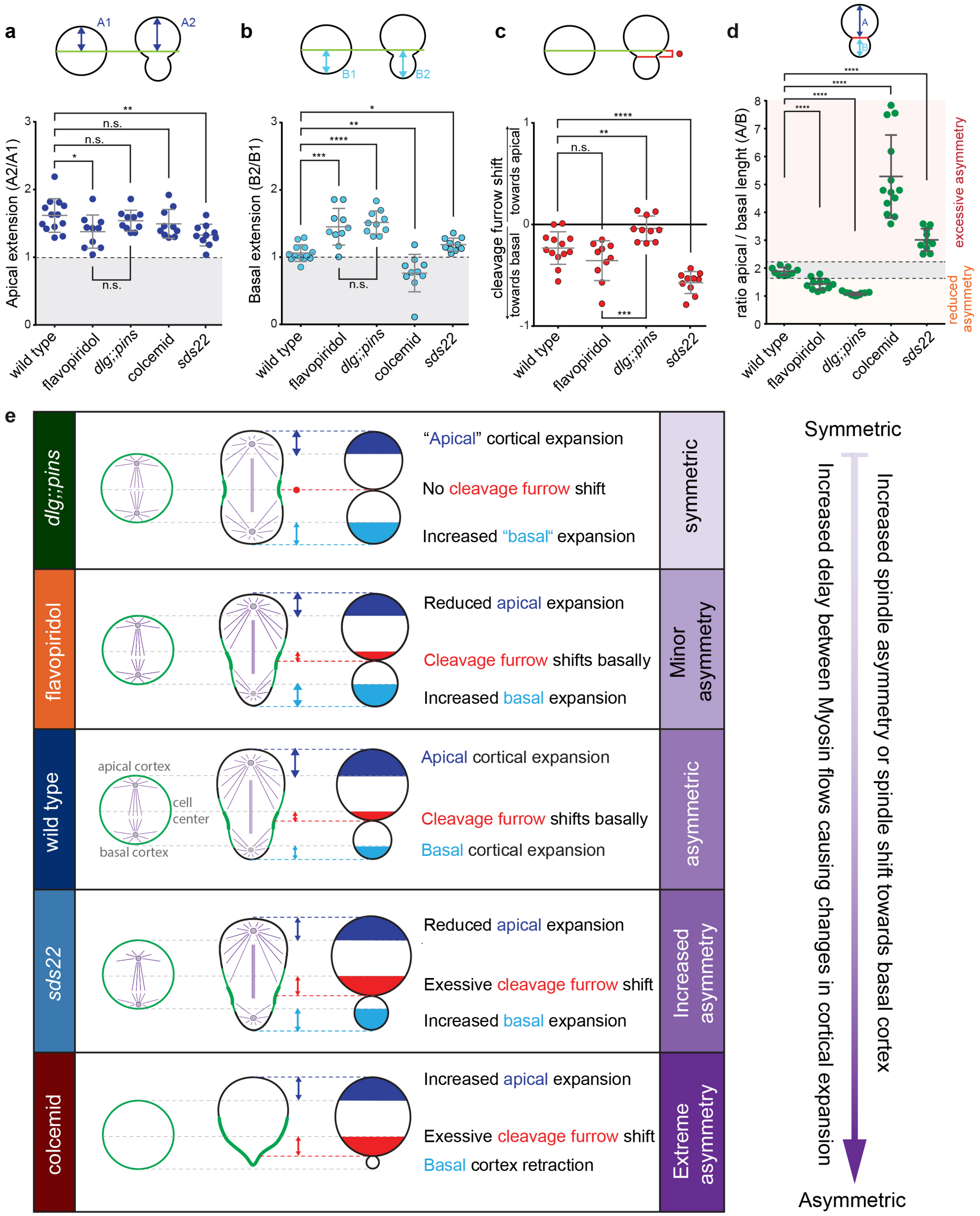

a
A1
A2
Apical extension (A2/A1)
n.s.
n.s.
*
**
wild type
flavopiridol
dlg;;pins
colcemid
sds22
b
B1
B2
Basal extension (B2/B1)
***
****
**
*
n.s.
c
cleavage furrow shift
towards apical
towards basal
n.s.
**
****
***
d
A
B
ratio apical / basal lenght (A/B)
excessive asymmetry
reduced asymmetry
e
dlg;;pins
"Apical" cortical expansion
No cleavage furrow shift
Increased "basal" expansion
symmetric
flavopiridol
Reduced apical expansion
Cleavage furrow shifts basally
Increased basal expansion
Minor asymmetry
wild type
apical cortex
cell center
basal cortex
Apical cortical expansion
Cleavage furrow shifts basally
Basal cortical expansion
asymmetric
sds22
Reduced apical expansion
Exessive cleavage furrow shift
Increased basal expansion
Increased asymmetry
colcemid
Increased apical expansion
Exessive cleavage furrow shift
Basal cortex retraction
Extreme asymmetry
Symmetric
Increased delay between Myosin flows causing changes in cortical expansion
Increased spindle asymmetry or spindle shift towards basal cortex
Asymmetric

**Supplementary Figure 8: Cortical extension and cleavage furrow shift contribute to physical asymmetry.**

Scatter plots showing **(a)** apical and **(b)** basal cortical extension in wild type, flavopiridol-treated, colcemid-treated, *dlg;;pins* and *sds22* mutant neuroblasts. **(c)** Scatter plot showing the site of cleavage furrow formation and **(d)** physical asymmetry for the indicated genotypes. **(e)** Summary of phenotypes. An increase in asymmetry can be obtained by shifting the spindle basally and/or increasing its asymmetry. Changes in cortical flow onset, affecting cortical expansion also contribute to increased or reduced physical sibling cell size asymmetry. Center values and error bars represent the mean and standard deviation (s.d), respectively. Asterisks denote statistical significance, derived from unpaired t-tests: *; $p \leq 0.05$, **; $p \leq 0.01$, ***; $p \leq 0.001$, ****; $P \leq 0.0001$, n.s.; not significant. Each measured cell (n) is represented with a dot in the scatter plots. For each experiment, the data was collected from at least 3 independent experiments. For each independent experiment, at least 5 larvae were dissected.
