# Supplementary material for: Spatio-temporally separated cortical flows and spindle geometry establish physical asymmetry in fly neural stem cells
Source: Nat Commun. 2017 Nov 9;8:1383. doi: 10.1038/s41467-017-01391-w (PMC5680339; doi:10.1038/s41467-017-01391-w)
Supplement: Supplementary file 3 — Description of Additional Supplementary Files [file 41467_2017_1391_MOESM3_ESM.pdf]

Description of Additional Supplementary Files

File Name: Supplementary Movie 1

Description: Apical Myosin filaments contribute to the cleavage furrow in telophase in intact larval fly brains.
Third instar larval wild type neuroblast of an intact brain lobe, expressing the photoconvertable fluorescent protein mDendra2, fused with Myosin's regulatory subunit, encoded by spaghetti squash (sqh::mDendra2; white). Myosin was photoconverted on the apical cortex. The movie starts with the first frame after photoconversion. The neuroblast was imaged every 30s. Time scale is h:mm:ss:ms. The scale bar is 5 µm.

File Name: Supplementary Movie 2

Description: Apical Myosin filaments contribute to the cleavage furrow in telophase in isolated neuroblasts.
Third instar larval wild type neuroblast from a primary culture expressing the photoconvertable fluorescent protein mDendra2, fused with Myosin's heavy chain, encoded by zipper (zip::mDendra2; white). Myosin was photoconverted on the apical cortex (purple box). Unconverted Myosin (white) is shown on the left, converted Myosin (white) in the middle and both unconverted (green) and converted Myosin (red) are shown on the right. The neuroblast was imaged every 9.9s. Time is in seconds. The scale bar is 5 µm.

File Name: Supplementary Movie 3

Description: Basal Myosin filaments contribute to the cleavage furrow in telophase in isolated neuroblasts.
Third instar larval wild type neuroblast from a primary culture expressing zip::mDendra2 (white). Myosin was photoconverted on the basal cortex (purple box). Unconverted Myosin (white) is shown on the left, converted Myosin (white) in the middle and both unconverted (green) and converted Myosin (red) are shown on the right. The neuroblast was imaged every 10s. Time is in seconds. The scale bar is 5 µm.

File Name: Supplementary Movie 4

Description: Myosin filaments reach the cleavage furrow through cortical flow.
Third instar larval wild type neuroblast of an intact brain lobe, expressing photoconvertable Sqh::mDendra2. Myosin was photoconverted laterally in early anaphase. Note that photoconverted Myosin remains confined to the lateral region and moves towards the cleavage furrow. The neuroblast was imaged every 0.43s before photoconversion and every 4.3s after photoconversion. Time scale is h:mm:ss:ms. The scale bar is 5 µm.

File Name: Supplementary Movie 5

Description: Apically photoconverted Gap43 spreads over the entire neuroblast cortex.
Isolated and cultured third instar larval wild type neuroblast expressing Gap43 fused to the photoconvertable fluorescent protein mEos (Gap43::mEos). Photoconversion was performed on the apical neuroblast cortex (purple box) and imaged every 9.6s. Unconverted Gap43 is shown on the left (white), converted Gap43 in the middle (white) and both unconverted (green) and converted (red) Gap43 to the right. Time is in seconds (s). The scale bar is 5 µm.

File Name: Supplementary Movie 6

Description: Laterally photoconverted Gap43 does not flow to the cleavage furrow.
Isolated and cultured third instar larval wild type neuroblast expressing Gap43::mEos. Photoconversion was performed on the lateral neuroblast cortex (purple box) and imaged every 10.5s. Note that photoconverted Gap43 does not remain confined to the lateral region but spreads all over the membrane. Unconverted Gap43 is shown on the left (white), converted Gap43 in the middle (white) and both unconverted (green) and converted (red) Gap43 to the right. Time is in seconds. The scale bar is 5 µm.

File Name: Supplementary Movie 7

Description: Myosin relocalizes to the cleavage furrow after lesion-induced cortical delocalization.
Third instar larval wild type neuroblast of an intact brain lobe, expressing Sqh::GFP (Myosin ; white) and imaged every 10s. A UV laser pulse was used to cut the cortex in anaphase. The yellow arrow refers to the laser-cutting site and the yellow box highlights the cortical lesion after the cut. Note that Myosin is completely removed from the neuroblast cortex after the cut but relocalizes to the cleavage furrow subsequently. Time is in seconds. The scale bar is 5 μm.

File Name: Supplementary Movie 8

Description: Spindle cues are not required to relocalize Myosin to the anaphase cortex but necessary for confined recruitment.
Third instar larval rod mutant, colcemid-treated neuroblast of an intact brain lobe, expressing Sqh::GFP (Myosin ; white) and imaged every 10s. A UV laser pulse was used to cut the cortex in anaphase (yellow arrow; yellow box). Note that Myosin is completely removed from the neuroblast cortex and is not restricted to the cleavage furrow when it relocalizes. Time is in seconds. The scale bar is 5 μm.

File Name: Supplementary Movie 9

Description: Retaining Myosin on the apical neuroblast cortex inverts physical asymmetry.
Representative third instar sqh mutant larval neuroblast, expressing ALD-RockCA::VhhGFP4, Sqh::GFP (Myosin; white on the left, green in the merged channel) and Cherry::Jupiter (MTs; red in the merged channel). Times is in seconds. The scale bar is 5 μm.
